# Correlation Between Early Endpoints and Overall Survival in Non-Small-Cell Lung Cancer: A Trial-Level Meta-Analysis

**DOI:** 10.3389/fonc.2021.672916

**Published:** 2021-07-26

**Authors:** Khader Shameer, Youyi Zhang, Dan Jackson, Kirsty Rhodes, Imran Khan A. Neelufer, Sreenath Nampally, Andrzej Prokop, Emmette Hutchison, Jiabu Ye, Vladislav A. Malkov, Feng Liu, Antony Sabin, Jim Weatherall, Cristina Duran, Renee Bailey Iacona, Faisal M. Khan, Pralay Mukhopadhyay

**Affiliations:** ^1^ Data Science and Artificial Intelligence, BioPharmaceuticals Research and Development (R&D), AstraZeneca, Gaithersburg, MD, United States; ^2^ Oncology Biometrics, Oncology Research and Development, AstraZeneca, Cambridge, United Kingdom; ^3^ Data Science and Artificial Intelligence, BioPharmaceuticals Research and Development, AstraZeneca, Macclesfield, United Kingdom; ^4^ Oncology Biometrics, Oncology Research and Development, AstraZeneca, Warsaw, Poland; ^5^ Digital Health, Oncology Research and Development, AstraZeneca, Cambridge, United Kingdom; ^6^ Oncology Biometrics, Oncology R&D, AstraZeneca, Gaithersburg, MD, United States

**Keywords:** surrogate endpoints, progression-free survival, correlation analysis, trial-level analysis, meta-regression analysis

## Abstract

Early endpoints, such as progression-free survival (PFS), are increasingly used as surrogates for overall survival (OS) to accelerate approval of novel oncology agents. Compiling trial-level data from randomized controlled trials (RCTs) could help to develop a predictive framework to ascertain correlation trends between treatment effects for early and late endpoints. Through trial-level correlation and random-effects meta-regression analysis, we assessed the relationship between hazard ratio (HR) OS and (1) HR PFS and (2) odds ratio (OR) PFS at 4 and 6 months, stratified according to the mechanism of action of the investigational product. Using multiple source databases, we compiled a data set including 81 phase II–IV RCTs (35 drugs and 156 observations) of patients with non-small-cell lung cancer. Low-to-moderate correlations were generally observed between treatment effects for early endpoints (based on PFS) and HR OS across trials of agents with different mechanisms of action. Moderate correlations were seen between treatment effects for HR PFS and HR OS across all trials, and in the programmed cell death-1/programmed cell death ligand-1 and epidermal growth factor receptor trial subsets. Although these results constitute an important step, caution is advised, as there are some limitations to our evaluation, and an additional patient-level analysis would be needed to establish true surrogacy.

## Introduction

In clinical trials that assess novel therapeutic agents in patients with non-small-cell lung cancer (NSCLC), overall survival (OS) is considered the gold-standard endpoint for establishing clinical benefit (1–3). ‘Early’ endpoints, such as progression-free survival (PFS) and objective response rate (ORR), are evaluated in oncology trials as indicators of biological drug activity. For example, PFS rate at 6 months (PFS6) is often used as the key endpoint in phase II trials to accelerate approval of novel therapies (3–5). Approximately two-thirds of all regulatory approvals for cancer drugs in the US are based on these surrogate endpoints, which also form the basis of early go/no go decisions in the drug development pipeline (for instance, the decision to initiate phase III trials) (1–3, 6). This is because they permit shorter trial durations and the use of smaller patient cohorts, thereby allowing for faster, more cost-effective trials (3, 6). The use of early endpoints can overcome certain limitations associated with using OS, including the impact of subsequent therapy and patient crossover between trial arms (7). Analyses to support the use of these early endpoints in oncology trials has also been extended to evaluating PFS as a surrogate endpoint for health-related quality of life (8–10).

Surrogate endpoints are a measure of the treatment effect that correlates with OS, the long-term, established clinical endpoint (11). To be a reliable substitute for OS, regulatory agencies require that these early endpoints follow the pattern of the late endpoint both as an epidemiological marker and as a therapeutic responder (11–13).

Using early endpoints as surrogates for OS has the potential to be misleading in terms of treatment benefit (14, 15). Previous analyses have not always demonstrated a clear relationship between these endpoints, and the correlation of early endpoints with OS across clinical trials of anti-cancer drugs with different mechanisms of action (MoA) is not well established (2, 16–18). This is important when considering the high failure rate of oncology trials in general, and phase III trials in particular, which is largely due to a failure to meet the primary efficacy endpoint and is associated with high human and financial costs (19–21).

Compiling trial-level data from randomized controlled trials (RCTs) could help to develop a predictive framework to ascertain the correlation trends between treatment effects for early (e.g., odds ratio [OR] for PFS at 4 or 6 months [PFS4 or PFS6]) and late (e.g., hazard ratio [HR] OS) endpoints in clinical trials. In turn, this could improve early go/no go decision making in the drug development pipeline, optimize the selection of early endpoints, constitute a first step towards establishing surrogacy of early endpoints for OS, and support payer recognition of PFS for reimbursement. Here, we compile trial-level data from RCTs of NSCLC and use the data set to evaluate correlations between treatment effects for early endpoints (based on PFS) and HR OS for all trials and stratified according to the MoA of the investigational product.

## Methods

### Systematic Literature Review

A trial-level data set was compiled, which included phase II–IV RCTs of Stages I–IV NSCLC (**Supplementary Figure 1**). The data set was collected from multiple source databases, namely Citeline’s Trial Trove, clinicaltrials.gov, PubMed, and an internal AstraZeneca database (constrained search). First, an initial list of trials was compiled based on Trialtrove and clinicaltrials.gov, with search restricted to between January 2000 and January 2019, using the following search terms: non-small-cell lung cancer/NSCLC (disease); phase II to phase IV (to identify randomized controlled trials). Additional evidence was extracted from PubMed for external publications (using PubMed ID numbers), and from the internal AstraZeneca database for clinical study reports on AstraZeneca trials. The search strategy for compiling the data set included considerations of whether PFS (assessed by blinded independent central review or by the investigator per Response Evaluation Criteria in Solid Tumors [RECIST]) and OS data were available, the trial was interventional/multi-arm, and the data were analysis-ready. Trials that did not have a full data set (i.e., HR OS and HR PFS data) were excluded. Between-trial biases (e.g., crossover, differences in length of follow-up, etc.) and attrition rates were not considered as part of the inclusion/exclusion criteria.

### Meta-Analysis: Data Extraction

The following treatment effect estimates were extracted from the identified trial reports, where available (reported HRs per Cox regression): HR OS, HR PFS, OR PFS4, and OR PFS6. ORs for PFS4 and PFS6 were calculated by extracting information from the curated data on how many patients had/did not have progression at 4 or 6 months, respectively, in the investigational arm and the control arm (attained by data mining of the reported Kaplan–Meier curves using the ‘WebPlotDigitizer’ tool (22) and establishing a contingency table based on these data (using actual count values). Fisher’s exact tests were then used to calculate ORs in a manner similar to computing ORs based on the proportion of PFS directly but using conditional maximum likelihood estimator (MLE) rather than unconditional MLE.

### Meta-Analysis: Correlation and Meta-Regression Analysis

Correlation and random-effects meta-regression analyses were carried out to assess relationships between HR OS and HR PFS, OR PFS4, and OR PFS6 across all trials and stratified according to the MoA of the investigational product. Spearman’s rank correlation coefficients (Rho) were derived for all comparisons between trial-level treatment effects; an absolute value of a correlation (Spearman’s rho) close to 1 (for HR vs HR comparisons) or –1 (for HR vs OR comparisons) indicated a strong monotonic association. Associations were categorized as very high (0.9 – 1.0); high (0.7 – <0.9); moderate (0.5 – <0.7); low (0.3 – <0.5); and negligible (0 – <0.3), as used previously (23). Trial-level associations were quantified through random-effects meta-regression. R^2^ was used to quantify the proportion of heterogeneity accounted for by the regression (restricted maximum likelihood method) using the “metafor” R package (24); log HR was used to decrease the effect of outliers and support the normality assumptions made by meta-regression models. Meta-regression analyses were performed across different data-strata, stratified by the MoA of the investigational product.

## Results

### Literature Search Results and Data Selection for Downstream Analysis

**Figure 1:**
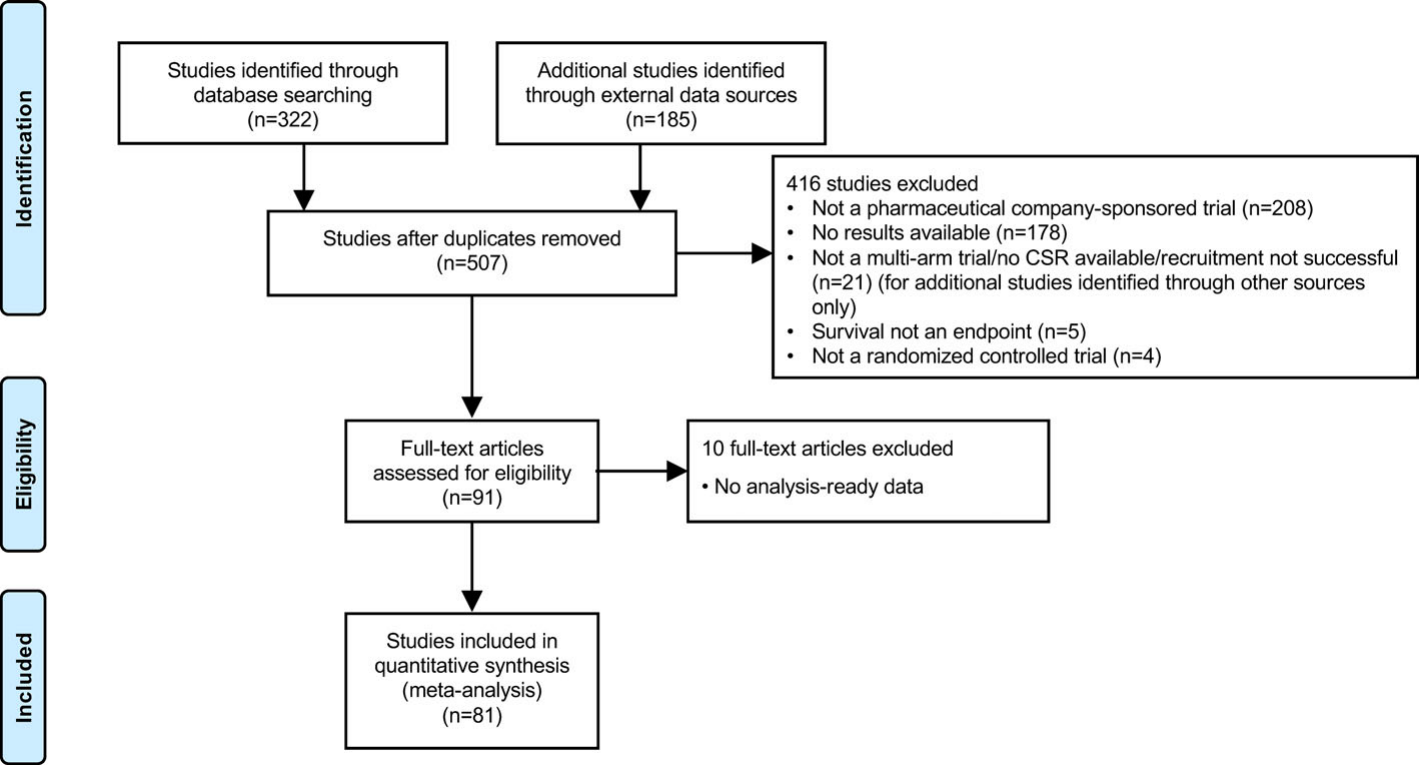
PRISMA flow diagram. CSR, clinical study report.

In total, the data set included 81 industry-wide RCTs with 35 drugs and 156 observations, as shown in the PRISMA flow diagram (**Figure 1**, **Supplementary Table 1**). Among the 15 different MoA groups identified in these trials, epidermal growth factor receptor (EGFR) inhibition constituted the largest group, with 25 trials included, followed by programmed cell death-1/programmed cell death ligand-1 (PD-1/PD-L1) inhibition (18 trials), vascular endothelial growth factor receptor (VEGFR) inhibition (13 trials), and DNA damage response (DDR) inhibition (six trials). These four major trial subsets were used for downstream analysis by MoA. Other MoAs included in the data set were as follows: tubulin inhibition (four trials); anaplastic lymphoma kinase (ALK) inhibition (four trials); mitogen-activated protein kinase (MEK) inhibition (three trials); and one trial each for inhibition of Toll-like receptor-9 (TLR-9), poly ADP ribose polymerase (PARP), thymidylate synthase (TYMS), insulin-like growth factor-1 receptor (IGF-1R), cytotoxic T-lymphocyte-associated protein-4 (CTLA-4), matrix metalloproteinase (MMP), and mucin-1 (MUC-1). The MoA was not available for one of the trials. Approximately 16% of trials allowed crossover, mostly in trials of PD-1/PD-L1 inhibitors.

### Trial-Level Correlation and Random-Effects Meta-Regression Analysis

#### HR OS *vs* HR PFS

**Figure 2:**
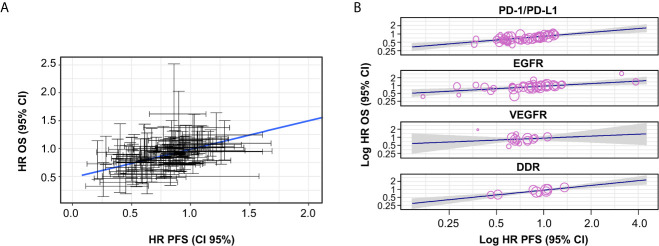
Correlation between HR OS and HR PFS, **(A)** across all trials and **(B)** by MoA. The gray-shaded area in panel **(B)** represents the pointwise 95% CI for the mean of the Y given X. CI, confidence interval; DDR, DNA damage response; EGFR, epidermal growth factor receptor; HR, hazard ratio; MoA, mechanism of action; OS, overall survival; PD-1/PD-L1, programmed cell death-1/programmed cell death ligand-1; PFS, progression-free survival; VEGFR, vascular endothelial growth factor receptor.

Based on 69 trials, a moderate correlation was observed between HR OS and HR PFS for all trials (i.e. irrespective of MoA) (random-effects meta-regression R^2^, 51.6%; *P* < 0.001) (**Figure 2A** and **Table 1**); the random-effect meta-regression Tau^2^ for between-trial variance was 0.034 (standard error, 0.008). Moderate correlations were also observed between HR OS and HR PFS for PD-1/PD-L1 inhibitors (random-effects meta-regression R^2^, 76.1%; *P* < 0.001) and EGFR inhibitors trials (random-effects meta-regression R^2^, 28.3%; *P* < 0.001) (**Figure 2B** and **Table 1**). The slopes were similar for PD-1/PD-L1 and EGFR inhibitors trials, but with different intercepts. The random-effects meta-regression R^2^ for EGFR inhibitors trials was small, suggesting that the regression fit was not reliable for this MoA. Negligible and high correlations were observed for VEGFR and DDR inhibitors, respectively, although these were based on very few observations (14 and 9, respectively) (**Figure 2B** and **Table 1**).

**Table 1:** Correlation between HR OS and HR PFS across all trials and by MoA.

Label	All trials	4 major MoAs combined	PD-1/PD-L1 inhibitors	EGFR inhibitors	VEGFR inhibitors	DDR inhibitors
Spearman’s Rho*	0.548	0.575	0.608	0.641	0.066	0.812
Spearman’s Rho 95% CI, bootstrap	(0.381; 0.689)	(0.404; 0.717)	(0.345; 0.801)	(0.368; 0.822)	(–0.557; 0.725)	(0.205; 1.000)
Number of drugs	32	20	5	6	5	4
Number of trials	69	54	17	21	10	6
Number of observations^†^	121	99	41	35	14	9
Slope, meta-regression	0.410 (0.303; 0.516)	0.423 (0.304; 0.541)	0.465 (0.291; 0.640)	0.322 (0.150; 0.495)	0.239 (–0.270; 0.749)	0.593 (0.367; 0.819)
Random-effects, meta-regression R^2^	51.59%	48.89%	76.06%	28.27%	0%	100%
P-value	<0.001	<0.001	<0.001	<0.001	0.357	<0.001

*The reported Rho values are negative as an HR <1, and an OR >1, indicate benefit with the investigational product. ^†^Cohort level.

CI, confidence interval; DDR, DNA damage response; EGFR, epidermal growth factor receptor; HR, hazard ratio; MoA, mechanism of action; OS, overall survival; PD-1/PD-L1, programmed cell death-1/programmed cell death ligand-1; PFS, progression-free survival; VEGFR, vascular endothelial growth factor receptor.

#### HR OS *vs* OR PFS 4/6 Months

Based on 64 trials, low correlations were observed between both HR OS and OR PFS4 (random-effects meta-regression R^2^, 10.9%; P < 0.001) and HR OS and OR PFS6 (random-effects meta-regression R^2^, 23.1%; P < 0.001) for all trials. The meta-regression R^2^ was small, suggesting that the regression fit was not reliable (**Supplementary Figures 2** and **3** and **Supplementary Tables 2** and **3**).

**Figure 3:**
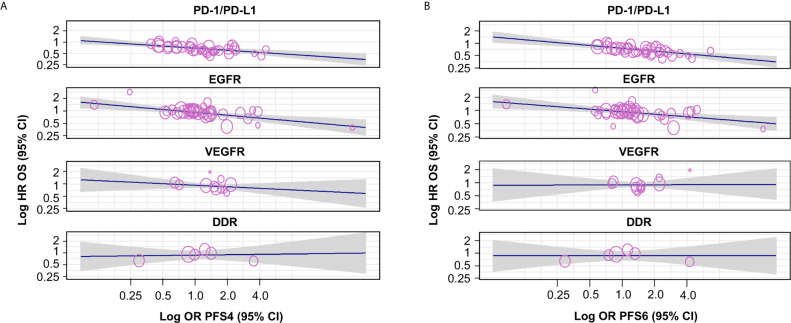
Correlation by MoA between HR OS and **(A)** OR PFS4 and **(B)** OR PFS6. The gray-shaded area in panels **(A, B)** represents the pointwise 95% CI for the mean of the Y given X. The reported Rho values are negative as an HR <1, and an OR >1, indicating benefit with the investigational agent. CI, confidence interval; DDR, DNA damage response; EGFR, epidermal growth factor receptor; HR, hazard ratio; MoA, mechanism of action; OR, odds ratio; OS, overall survival; PD-1/PD-L1, programmed cell death-1/programmed cell death ligand-1; PFS4/6, progression-free survival rate at 4/6 months; VEGFR, vascular endothelial growth factor receptor.

Moderate correlations were observed between HR OS and OR PFS4 for PD-1/PD-L1 inhibitors (random-effects meta-regression R^2^, 72.5%; P < 0.001) and EGFR inhibitors trials (random-effects meta-regression R^2^, 35.6%; P < 0.001) (**Figure 3** and **Table 2**). Similar correlations to those observed between HR OS and OR PFS4 were observed between HR OS and OR PFS6 for PD-1/PD-L1 inhibitors (random-effects meta-regression R^2^, 86.1%; P < 0.001) and EGFR inhibitors trials (random-effects meta-regression R^2^, 36.2%; P < 0.001) (**Figure 3** and **Table 2**). The slopes were similar for PD-1/PD-L1 and EGFR inhibitors trials, but with different intercepts. The random-effects meta-regression R^2^ for EGFR inhibitors trials was small, suggesting that the regression fit was not reliable for this MoA. For VEGFR and DDR inhibitors trials, negligible to low correlations were observed between both HR OS and OR PFS4 and HR OS and OR PFS6, although these were based on very few observations (11 and 6, respectively) (**Figure 3** and **Table 2**).

**Table 2:** Correlation by MoA between HR OS and OR PFS4/OR PFS6.

Correlations with HR OS								
	PD-1/PD-L1 inhibitors		EGFR inhibitors		VEGFR inhibitors		DDR inhibitors	
	OR PFS4	OR PFS6	OR PFS4	OR PFS6	OR PFS4	OR PFS6	OR PFS4	OR PFS6
Spearman’s Rho*	–0.579	–0.633	–0.535	–0.427	–0.443	0.224	0.029	0.086
Spearman’s Rho 95% CI, bootstrap	(–0.800;–0.274)	(–0.802;–0.383)	(–0.760;–0.230)	(–0.705;–0.085)	(–0.993; 0.146)	(–0.638; 0.795)	(–1.000; 1.000)	(–0.920; 1.000)
Number of drugs	5	5	6	6	5	5	4	4
Number of trials	16	16	21	21	8	8	5	5
Number of observations^†^	38	38	37	37	11	11	6	6
Slope, meta-regression	–0.192 (–0.280;–0.104)	–0.229 (–0.321;–0.136)	–0.230 (–0.344;–0.116)	–0.191 (–0.297;–0.086)	–0.125 (–0.356; 0.106)	0.007 (–0.269; 0.283)	0.033(–0.285; 0.352)	0 (–0.299; 0.299)
Random-effects, meta-regression R^2^	72.48%	86.13%	35.63%	36.17%	0%	0%	0%	0%
P-value	< 0.001	< 0.001	< 0.001	< 0.001	0.289	0.959	0.838	0.999

*The reported Rho values are negative as an HR <1, and an OR >1, indicate benefit with the investigational product. ^†^Cohort level.

CI, confidence interval; DDR, DNA damage response; EGFR, epidermal growth factor receptor; HR, hazard ratio; MoA, mechanism of action; OR, odds ratio; OS, overall survival; PD-1/PD-L1, programmed cell death-1/programmed cell death ligand-1; PFS4/6, progression-free survival rate at 4/6 months; VEGFR, vascular endothelial growth factor receptor.

**Figure 4:**
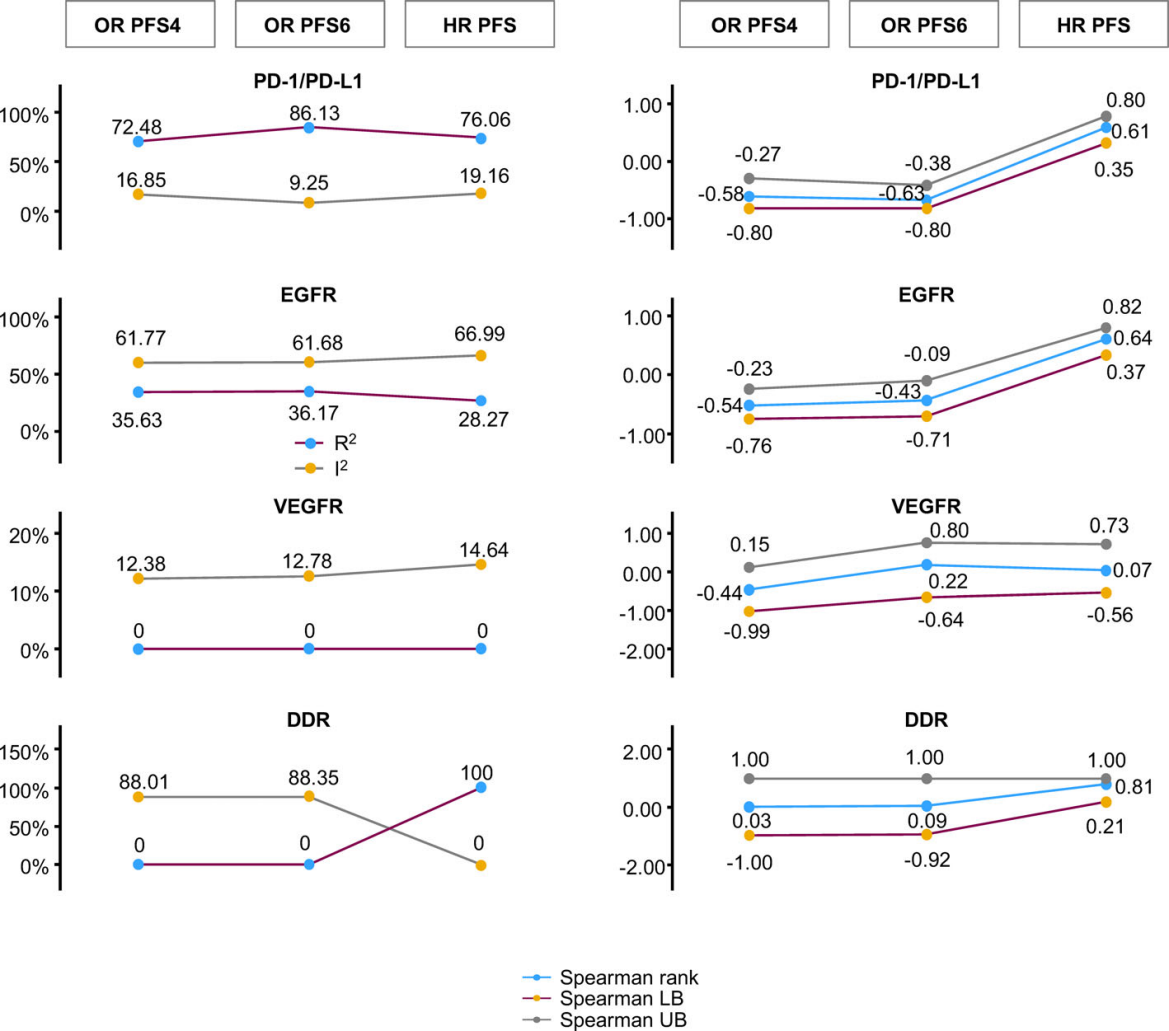
Changes in random-effects meta-regression R^2^ and I^2^, Spearman’s rho, and Spearman’s rho upper/lower bound 95% CI for HR OS versus PFS-based treatment effects. The statistics for random-effects meta-regression for OR PFS at 4 months, OR PFS at 6 months, and HR PFS are compared with HR OS in a single plot. This represents the comparison of random-effects meta-regression R^2^ and I^2^ on the left, and Spearman’s rank correlation at 95% CI bootstrap with its upper bound and lower bound on the right. CI, confidence interval; DDR, DNA damage response; EGFR, epidermal growth factor receptor; HR, hazard ratio; LB, lower bound; OR, odds ratio; OS, overall survival; PD-1/PD-L1, programmed cell death-1/programmed cell death ligand-1; PFS, progression-free survival; PFS4/6, progression-free survival rate at 4/6 months; UB, upper bound; VEGFR, vascular endothelial growth factor receptor.

Changes in random-effects meta-regression R^2^, random-effects meta-regression I^2^, Spearman’s rho, and Spearman’s rho upper/lower bound 95% CI for HR OS versus the different PFS-based treatment effects are summarized in **Figure 4**.

## Discussion

Compiling trial-level data from oncology RCTs to ascertain correlation trends between treatment effects for early and late endpoints have the potential to improve early go/no go decision making in the drug development pipeline, optimize the selection of early endpoints, and support payer recognition of PFS for reimbursement, allowing for faster and more cost-effective oncology trials.

Using a comprehensive, trial-level summary data set of 35 drugs, 81 trials, and 156 observations in the NSCLC setting, we evaluated correlations between treatment effects for early endpoints (based on PFS) and HR OS. Low-to-moderate correlations were observed between HR PFS and HR OS across RCTs of agents with different MoAs. Trends were similar for PD-1/PD-L1 checkpoint inhibitors and EGFR inhibitors, although, in the latter case, the random-effects meta-regression R^2^ was small, suggesting that the regression fit was not reliable for this MoA. Moderate and low-to-moderate correlations, respectively, were also observed between treatment effects for OR PFS4/6 and HR OS in trials of PD-1/PD-L1 checkpoint inhibitors and EGFR inhibitors. These results suggest that, for these classes of agents, an improvement in OR PFS4/6 can be associated with OS benefit, and that PFS4 could potentially be used instead of PFS6 in early phase clinical trials, thereby speeding up the completion of these trials while providing support for initiating phase III trials.

The trial-level correlations also constitute a first step toward establishing the surrogacy of PFS for OS, although patient-level analyses would also be required for this purpose (25). Nevertheless, the results for PD-1/PD-L1 checkpoint inhibitor trials in the current analysis are broadly consistent with the results of another recent meta-analysis that assessed the surrogacy of PFS for OS with PD-1/PD-L1 checkpoint inhibitors at both the trial and patient level; in the trial-level analysis, based on 40 RCTs across various solid tumors, high correlation was observed between HR PFS and HR OS, with modest or limited benefit in PFS associated with meaningful improvement in OS (23). In the patient-level analysis, a positive association was observed between PFS and OS in NSCLC (Kendall’s Tau, 0.793; 95% CI, 0.789–0.797), as well as in other solid tumors, such as head and neck squamous cell carcinoma and bladder cancer. However, modest or limited improvement in RECIST-based endpoints did not rule out meaningful OS benefit, suggesting that they are imperfect surrogates that do not fully capture the clinical benefit of PD-1/PD-L1 checkpoint inhibitors (23). This warrants caution when basing early discontinuation of novel agents in this class on these surrogate endpoints.

Another meta-regression analysis of trials in patients with NSCLC provided no evidence of trial-level correlations (meta-regression R^2^, 0.08; 95% CI, 0–0.31) between treatment effects for PFS and OS for targeted therapies, such as EGFR inhibitors (16). In the current analysis, a moderate correlation was observed between treatment effects for HR OS and HR PFS for EGFR inhibitor trials, although the random-effects meta-regression R^2^ was small, suggesting that the regression fit was not reliable for this MoA. Taken together, these results suggest that PFS is an imperfect surrogate for OS in trials of EGFR inhibitors.

An analysis of 60 RCTs in patients with lung cancer assessed in six meta-analyses showed that PFS was a valid surrogate endpoint for OS in trials of chemotherapy and radiotherapy for patients with locally advanced lung cancers at trial level (R^2^ range, 0.89–0.97) (18).

Analyses of trials assessing anti-angiogenic agents and EGFR inhibitors in first-line metastatic colorectal cancer showed modest correlations between PFS and OS (R^2^ range, 0.45–0.69) (26). A trial-level meta-analysis of the correlation between PFS and OS in trials assessing chemotherapy or targeted therapy in metastatic breast cancer showed that HR PFS was a significant predictor of HR OS; however, when assessing by line of therapy, the association was significant in second-line and beyond trials, but not in the first-line trials (27). In the current analysis, no evaluation was conducted by line of therapy (first-line versus second-line and beyond), and it is therefore not possible to conclude whether there were any differences by line of therapy. The current analysis is also limited by the fact that the studies included were in the NSCLC setting only; it cannot be assumed that similar results would be observed with other cancers.

Moreover, the analysis was not stratified by the stage of disease under study, the nature of the control arm, the length of follow-up, or the line of therapy, potentially confounding the results. Regarding the different stages of disease included in the analysis, it is worth noting that all studies were in the locally advanced/advanced setting (stage III/IV), with no studies in patients with stage I/II disease and a majority of studies (62/81) in patients with stage IIIB/IV disease (with an additional 14 studies in patients with stage IV disease, two in patients with stage III/IV disease, one in patients with stage IIIA/B disease, and two in patients with stage III disease). Therefore, the results of this analysis largely reflect the locally advanced/advanced setting. An analysis by stage of disease would be of interest in follow-up investigations to assess any potential differences between early-stage and late-stage disease. Inclusion of different lines of therapy in the analysis is also a limitation, with inclusion of 46 trials in the first-line setting, and 31 in the second-line and above setting (four not available). Because the treatment intent is different for first-line versus further lines of therapy, an analysis by line of therapy would be of interest in follow-up investigations. Finally, the inclusion of studies with different lengths of follow-up is a common challenge in meta-analyses (28); a limitation of this analysis is the method commonly used for pooling of data when follow-up duration variables were not used.

Although the approach used to extract the data is reproducible, the specific extracted data points for PFS at 4 and 6 months may deviate in value, as these data were obtained through data mining of Kaplan-Meier curves. Additional limitations include cross-mechanism grouping; trial outcomes being closer to one for HR OS; and the studies included in the analysis being a heterogeneous mix of MoAs and study designs, with some studies pre-dating 2010 [i.e., before the first trials of PD-1/PD-L1 checkpoint inhibitors in NSCLC (29)]. As a result of these additional limitations, the correlation might have been more or less pronounced in analyses stratified by MoA, compared with combined analyses. Phase II or crossover studies were also considered in the modeling, with approximately 16% of trials allowing crossover; based on a separate analysis of crossover, it is thought, however, that this should not have affected the results significantly. In addition, inclusion of phase II studies could have also impacted the results. However, only 13 of 81 trials included in the analysis were phase II trials (plus 1 phase II/III trial and 1 phase IV trial), and 66 of 81 studies were phase III trials; therefore, the results largely reflect phase III trials. Conclusions cannot be drawn for the VEGFR and DDR inhibitors trial subsets because of the low number of observations; this is because when estimation methods are based on asymptotical assumptions, they can easily be biased when the sample size is small, and a recommendation is that meta-regression should generally not be considered when there are fewer than 10 studies available (30).

For this analysis, we also decided to only assess trial-level correlations and use a systematic approach largely based on the clinicaltrials.gov database, with searches carried out over 18 months. In this approach, not all studies are reported, and some studies only provide partial information or are ongoing. However, even when the treatment has a positive impact on the early endpoint and the early endpoint and OS are positively correlated, it is still possible that the treatment has no impact or a negative impact on OS, which challenges the use of surrogate endpoints. Therefore, because of the nature of a trial-level analysis, when assessing the validity of a surrogate, it is important to consider potential confounding factors and whether it is possible for the treatment to affect the early endpoint for different patients than those for whom the early endpoint affects OS.

Following this trial-level analysis, other trial-level parameters could be built into a digital health aid, including different tumor types, additional early endpoints, such as ORR, and other non-RECIST-based endpoints, to continue building a predictive framework that may help to ascertain the correlation trends across early-to-late endpoints in clinical trials and reduce the failure rate of pivotal phase III trials (20, 21). The challenges of early-phase study design of immunotherapies require new approaches that include incorporating additional endpoints, for instance, in the dose selection process, to improve efficacy and reduce toxicity (31). In recent years, there have been calls for more widespread use of data-driven tools to augment shared decision making, to incorporate the patient perspective and increase trial participation (32), and to address issues associated with the conduct of randomized clinical trials during pandemics (33).

Furthermore, high-quality real-world evidence (RWE) could be leveraged to enable drug approvals in oncology (34, 35), linking it to the value proposition of drugs (36–38). Regulatory bodies, such as the US Food and Drug Administration, have recently shown a willingness to expedite access to new cancer medicines by using RWE (39).

## Conclusions

Using a comprehensive, trial-level, summary data set in the NSCLC setting, we generally observed low-to-moderate correlations between treatment effects for early endpoints (based on PFS) and HR OS across trials of agents with different MoAs. Moderate correlations were observed among trials of PD-1/PD-L1 checkpoint inhibitors and EGFR inhibitors. Caution is advised when drawing on the surrogacy of early endpoints for OS based on the current analysis, as an additional patient-level analysis would be needed to establish true surrogacy, and there are several limitations to the analysis. Exploration of additional endpoints, beyond RECIST, is needed to identify other early indicators of efficacy that might better predict HR OS. Moreover, compiling trial-level data for other solid tumors is required to optimize the selection of early endpoints across different cancer indications. By incorporating additional trial-level parameters and building composite biomarkers using machine intelligence methods, in collaboration with innovative trial design efforts, we envisage improving the prediction of HR OS from early endpoints.

## Data Availability Statement

The original contributions presented in the study are included in the article/**Supplementary Material**. Further inquiries can be directed to the corresponding author.

## Author Contributions

KS developed the project and analytics strategy with critical inputs from RI, CD, FMK, PM, and JW. YZ, VAM, SN, and EH led data compilation. AS, DJ, KR, IKAN, JY, FL, and KS contributed to the statistical analyses. IKAN conducted analyses. The original manuscript was written by KS, with additions to the manuscript and edits provided by YZ, DJ, KR, SN, AP, EH, JY, VM, FL, AS, JW, CD, RI, FMK, and PM. All authors contributed to the article and approved the submitted version.

## Funding

This study was funded by AstraZeneca.

## Conflict of Interest

KR, EH, FL, AS, JW, RI, and FK are full-time employees of AstraZeneca and own AstraZeneca stock. SK, YZ, DJ, SN, AP, JY, and CD are full-time employees of AstraZeneca. PM was a full-time employee of AstraZeneca at the time that the study was conducted and owns AstraZeneca stock. IK and VM were full-time employees of AstraZeneca at the time that the study was conducted.

The authors declare that this study received funding from AstraZeneca. The funder had the following involvement with the study: Study design and concept, collection, analysis and interpretation of the data, review and approval of the final draft and approval to submit for publication.

## Publisher’s Note

All claims expressed in this article are solely those of the authors and do not necessarily represent those of their affiliated organizations, or those of the publisher, the editors and the reviewers. Any product that may be evaluated in this article, or claim that may be made by its manufacturer, is not guaranteed or endorsed by the publisher.

## Supplementary Material

Supplementary Figure 1 Clinical trial search strategy. An initial list of trials was extracted from clinicaltrials.gov (left column) and Trialtrove (right column). The category ‘additional evidence from publications (external)’ indicates additional evidence extracted through PubMed; ‘additional evidence from CSRs (AZ)’ indicates additional evidence extracted through the internal AstraZeneca database. AZ, AstraZeneca; CSR, clinical study report; NSCLC, non-small-cell lung cancer; OS, overall survival; PFS, progression-free survival; Ph, phase; RCT, randomized controlled trial.

Supplementary Figure 2 Correlation across all trials and by MoA between HR OS and OR PFS4. The gray-shaded area in the figure represents the pointwise 95% CI for the mean of the Y given X. The reported Rho values are negative as an HR <1, and an OR >1, indicate benefit with the investigational agent. CI, confidence interval; DDR, DNA damage response; EGFR, epidermal growth factor receptor; HR, hazard ratio; MoA, mechanism of action; OR, odds ratio; OS, overall survival; PD-1/PD-L1, programmed cell death-1/programmed cell death ligand-1; PFS4, progression-free survival rate at 4 months; VEGFR, vascular endothelial growth factor receptor.

Supplementary Figure 3 Correlation across all trials and by MoA between HR OS and OR PFS6. The gray-shaded area in the figure represents the pointwise 95% CI for the mean of the Y given X. The reported Rho values are negative as an HR <1, and an OR >1, indicate benefit with the investigational agent. CI, confidence interval; DDR, DNA damage response; EGFR, epidermal growth factor receptor; HR, hazard ratio; MoA, mechanism of action; OR, odds ratio; OS, overall survival; PD-1/PD-L1, programmed cell death-1/programmed cell death ligand-1; PFS6, progression-free survival rate at 6 months; VEGFR, vascular endothelial growth factor receptor.
