# Supplementary figures and images for: Correlation Between Early Endpoints and Overall Survival in Non-Small-Cell Lung Cancer: A Trial-Level Meta-Analysis
Source: Front Oncol. 2021 Jul 26;11:672916. doi: 10.3389/fonc.2021.672916 (PMC8351517; doi:10.3389/fonc.2021.672916)

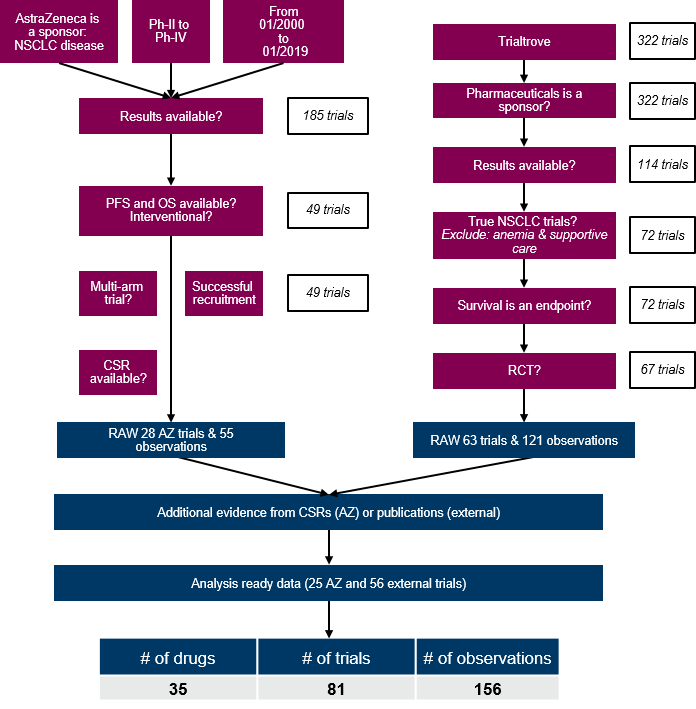

Supplement: Supplementary Figure 1 — Clinical trial search strategy. An initial list of trials was extracted from clinicaltrials.gov (left column) and Trialtrove (right column). The category ‘additional evidence from publications (external)’ indicates additional evidence extracted through PubMed; ‘additional evidence from CSRs (AZ)’ indicates additional evidence extracted through the internal AstraZeneca database. AZ, AstraZeneca; CSR, clinical study report; NSCLC, non-small-cell lung cancer; OS, overall survival; PFS, progression-free survival; Ph, phase; RCT, randomized controlled trial. [file Image_1.tif]

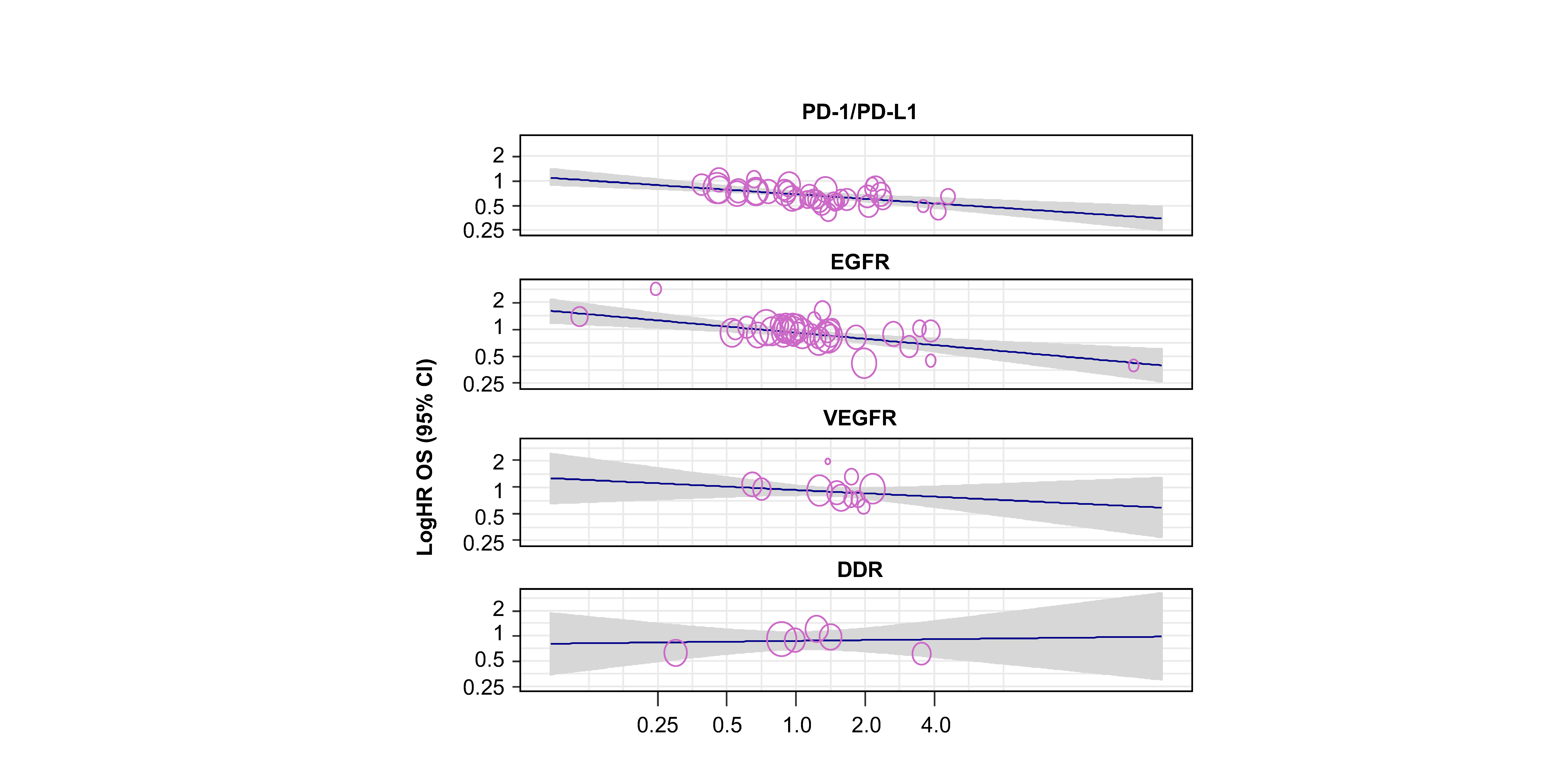

Supplement: Supplementary Figure 2 — Correlation across all trials and by MoA between HR OS and OR PFS4. The gray-shaded area in the figure represents the pointwise 95% CI for the mean of the Y given X. The reported Rho values are negative as an HR <1, and an OR >1, indicate benefit with the investigational agent. CI, confidence interval; DDR, DNA damage response; EGFR, epidermal growth factor receptor; HR, hazard ratio; MoA, mechanism of action; OR, odds ratio; OS, overall survival; PD-1/PD-L1, programmed cell death-1/programmed cell death ligand-1; PFS4, progression-free survival rate at 4 months; VEGFR, vascular endothelial growth factor receptor. [file Image_2.tif]

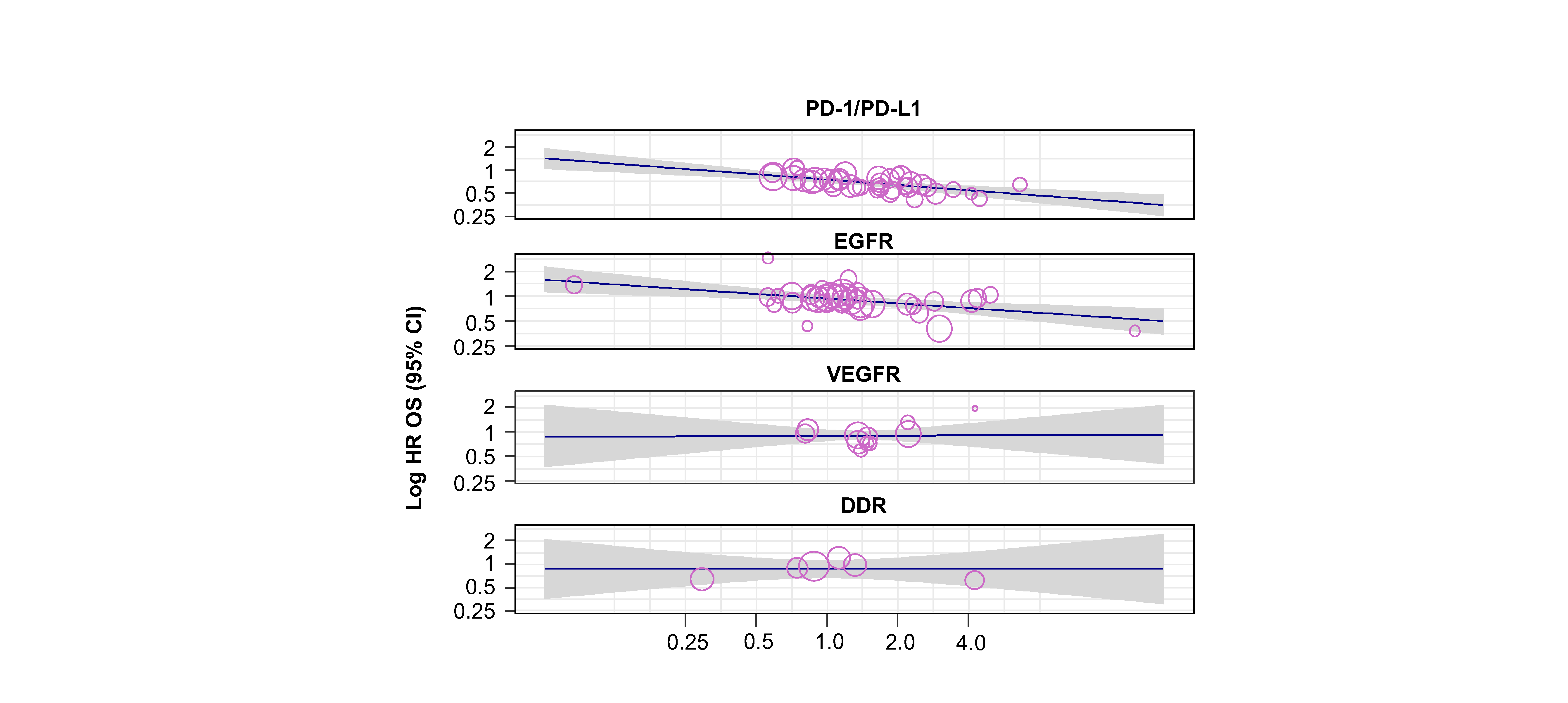

Supplement: Supplementary Figure 3 — Correlation across all trials and by MoA between HR OS and OR PFS6. The gray-shaded area in the figure represents the pointwise 95% CI for the mean of the Y given X. The reported Rho values are negative as an HR <1, and an OR >1, indicate benefit with the investigational agent. CI, confidence interval; DDR, DNA damage response; EGFR, epidermal growth factor receptor; HR, hazard ratio; MoA, mechanism of action; OR, odds ratio; OS, overall survival; PD-1/PD-L1, programmed cell death-1/programmed cell death ligand-1; PFS6, progression-free survival rate at 6 months; VEGFR, vascular endothelial growth factor receptor. [file Image_3.tif]
