# Supplementary material for: Correlation Between Early Endpoints and Overall Survival in Non-Small-Cell Lung Cancer: A Trial-Level Meta-Analysis
Source: Front Oncol. 2021 Jul 26;11:672916. doi: 10.3389/fonc.2021.672916 (PMC8351517; doi:10.3389/fonc.2021.672916)
Supplement: Supplementary file 4 [file Table_1.docx]

Supplementary Materials

Supplementary Table 1 | Literature search results for evaluation of trial-level correlations.

| **Study name** | **NCT#** | **Phase** | **NSCLC disease stage** | **MoA** | **Drug** | **Line of therapy** | **Nature of control arm** | **Crossover permitted (Y/N)** | **Population size** | **HR OS  (95% CI)** | **HR PFS**  **(95% CI)** | **% of patients with PFS at 4 months** | | **% of patients with PFS at 6 months** | |
| --- | --- | --- | --- | --- | --- | --- | --- | --- | --- | --- | --- | --- | --- | --- | --- |
|  |  |  |  |  |  |  |  |  |  |  |  | **ID** | **Crl** | **ID** | **Crl** |
| AURA3 (1) | NCT02151981 | III | IV | EGFR TKI | Osimertinib | 2L+ | Platinum-pemetrexed | Y | FAS  ID = 279  Crl = 140 | NA | 0.28  (0.20 – 0.38) | 83.1 | 61.8 | 69.7 | 34.2 |
|  |  |  |  |  |  |  |  |  | CNS mets  ID = 75  Crl = 41 | NA | 0.32  (0.21 – 0.49) | 89.9 | 77.0 | 75.1 | 44.5 |
| BR29 (2) | NCT00795340 | III | IIIB/IV | VEGFR TKI | Cediranib + carboplatin-paclitaxel | 1L | Paclitaxel-carboplatin | N | ID = 153 Crl + 153 | 0.95  (0.69 – 1.32) | 0.88 (0.68 – 1.14) | 67.1 | 74.2 | 37.6 | 43.3 |
| CAURAL (3) | NCT02454933 | III | IIIB/IV | PD-L1 inhibitor | Osimertinib +  durvalumab | 2L+ | Osimertinib | N | ID = 17  Crl = 12 | NA | NA | NA | NA | NA | NA |
| FLAURA (4) | NCT02296125 | III | IIIB/IV | EGFR TKI | Osimertinib | 1L | Gefitinib/ erlotinib | Y | FAS  ID = 279  Crl = 277 | 0.63  (0.45 – 0.88) | 0.46  (0.37– 0.57) | 95.6 | 87.8 | 88.1 | 75.2 |
|  |  |  |  |  |  |  |  |  | CNS mets  ID = 53  Crl = 63 | NA | 0.47  (0.30 – 0.74) | 93.4 | 87.0 | 80.1 | 65.1 |
|  |  |  |  |  |  |  |  |  | No CNS mets  ID = 226  Crl = 214 | NA | 0.46  (0.36 – 0.59) | 96.5 | 88.3 | 90.0 | 78.1 |
| IMPRESS (5) | NCT01544179 | III | IIIB/IV | EGFR TKI | Gefitinib + cisplatin-pemetrexed | 2L+ | Cisplatin-pemetrexed | N | ID = 133 Crl = 132 | 1.62 (1.05 – 2.52) | 0.86 (0.65 – 1.13) | 73.1 | 67.8 | 40.6 | 35.9 |
| INFORM (6) | NCT00770588 | III | IIIB/IV | EGFR TKI | Gefitinib | 2L+ | PBO | N | ITT  ID = 148  Crl = 148 | 0.88 (0.68–1.14) | 0.42 (0.32–0.54) | 56.2 | 32.7 | 47.8 | 18.1 |
|  |  |  |  |  |  |  |  |  | EGFR+ ID = 15  Crl = 15 | 0.39 (0.15–0.97) | 0.17 (0.07–0.42) | 93.2 | 26.7 | 86.7 | 20.0 |
|  |  |  |  |  |  |  |  |  | EGFR– ID = 25  Crl = 24 | 1.27 (0.70–2.30) | 0.86 (0.48–1.51) | 24.1 | 20.7 | 12.2 | 12.2 |
| INSTEP (7) | NCT00259064 | II | IIIB/IV | EGFR TKI | Gefitinib + BSC | 1L | PBO + BSC | N | ITT  ID = 100  Crl = 101 | 0.84  (0.62–1.15) | 0.82  (0.60–1.12) | 20.7 | 15.6 | 7.7 | 11.3 |
|  |  |  |  |  |  |  |  |  | EGFR+  ID = 12  Crl = 20 | 0.44  (0.17–1.12) | 0.29  (0.11–0.73) | 41.9 | 15.6 | 8.1 | 7.5 |
|  |  |  |  |  |  |  |  |  | EGFR-  ID = 32  Crl = 20 | 1.02  (0.56–1.88) | 0.74  (0.38–1.45) | 14.5 | 5.2 | 3.3 | 5.2 |
| INTACT1 (8) | NCT00006048 | III | IIIB/IV | EGFR TKI | Gefitinib  250 mg + gemcitabine-cisplatin | 1L | PBO + gemcitabine-cisplatin | N | ID = 365  Crl = 363 | 0.92  (0.77–1.10) | NA | 66.0 | 69.0 | 45.2 | 47.5 |
|  |  |  |  |  | Gefitinib  500 mg + gemcitabine-cisplatin |  | PBO + gemcitabine-cisplatin |  | ID = 365  Crl = 363 | 0.94  (0.77–1.12) | NA | 63.3 | 69.0 | 43.7 | 47.5 |
| INTACT2 (9) | NCT00006049 | III | IIIB/IV | EGFR TKI | Gefitinib  250 mg + paclitaxel-carboplatin | NA | PBO + paclitaxel-carboplatin | N | ID = 345  Crl = 345 | 1.03  (0.86–1.24) | 1.15  (0.96 – 1.38) | 59.8 | 60.6 | 37.7 | 36.9 |
|  |  |  |  |  | Gefitinib  500 mg + paclitaxel-carboplatin |  | PBO + paclitaxel-carboplatin |  | ID = 347  Crl = 345 | 0.96  (0.81–1.15) | 1.18  (0.97–1.43) | 59.8 | 60.9 | 36.5 | 36.9 |
| INVITE (10) | NCT00256711 | II | IIIB/IV | EGFR TKI | Gefitinib | 1L | Vinorelbine | N | FAS  ID = 97  Crl = 99 | 0.98  (0.66–1.47) | 1.19  (0.85–1.65) | 27.0 | 40.5 | 14.9 | 22.9 |
|  |  |  |  |  |  |  |  |  | EGFR+  ID = 30  Crl = 24 | 2.88  (1.21–6.83) | 3.13  (1.45–6.76) | 17.3 | 46.7 | 8.4 | 17.6 |
|  |  |  |  |  |  |  |  |  | EGFR–  ID = 47  Crl = 57 | 0.79  (0.46–1.37) | 0.93  (0.59–1.46) | 36.0 | 37.0 | 14.8 | 22.4 |
| IPASS (11) | NCT00322452 | III | IIIB/IV | EGFR TKI | Gefitinib | 1L | Carboplatin-paclitaxel | N | FAS  ID = 609  Crl = 608 | 0.91  (0.76–1.10) | 0.74  (0.65–0.85) | 58.8 | 73.1 | 48.1 | 48.2 |
|  |  |  |  |  |  |  |  |  | EGFR+  ID = 132  Crl = 129 | 0.78  (0.50–1.20) | 0.48  (0.34–0.67) | 81.1 | 77.2 | 69.6 | 49.7 |
|  |  |  |  |  |  |  |  |  | EGFR-  ID = 91  Crl = 85 | 1.38  (0.92–2.09) | 2.85  (2.05–3.98) | 22.6 | 72.7 | 5.1 | 41.7 |
|  |  |  |  |  |  |  |  |  | EGFR unknown  ID = 386  Crl = 394 | 0.86  (0.68–1.09) | 0.68  (0.58–0.81) | 59.9 | 68.8 | 50.2 | 46.3 |
| ISTANA (12) | NCT00478049 | III | IIIB/IV | EGFR TKI | Gefitinib | 2L+ | Docetaxel | N | ID = 82  Crl = 79 | 0.87  (0.61–1.24) | 0.63  (0.43–0.93) | 40.0 | 36.8 | 32.3 | 13.6 |
| N0528 (13) | NCT00326599 | II | IIIB/IV | VEGFR TKI | Cediranib 30 mg + gemcitabine-carboplatin | 1L | Gemcitabine/ carboplatin | N | ID = 58  Crl = 29 | 0.66  (0.41–1.08) | 0.69  (0.43–1.09) | 72.5 | 51.8 | 51.2 | 41.4 |
| INTEREST (14) | NCT00076388 | III | IIIB/IV | EGFR TKI | Gefitinib | 2L+ | Docetaxel | N | ITT  ID = 733  Crl = 733 | 1.01 (0.90–1.14) | 1.04  (0.93–1.18) | 33.8 | 40.9 | 21.6 | 33.8 |
|  |  |  |  |  |  |  |  |  | High EGFR copy number  ID = 85  Crl = 89 | 1.09 (0.78–1.51) | 0.84 (0.59–1.19) | 28.3 | 39.9 | 18.9 | 21.9 |
|  |  |  |  |  |  |  |  |  | Low EGFR copy number  ID = 93  Crl = 107 | 0.93  (0.68–1.26) | 1.30 (0.93–1.83) | 40.1 | 44.2 | 26.1 | 20.3 |
| V-15-32 (15) | NCT00252707 | III | IIIB/IV | EGFR TKI | Gefitinib | 2L+ | Docetaxel | N | ID = 245  Crl = 244 | 1.01  (0.80–1.27) | 0.81  (0.65–1.02) | 29.8 | 32.6 | 21.6 | 20.0 |
| NCT00777179 (16) | NCT00777179 | II | IIIB/IV | VEGFR TKI | Vandetanib + BSC | 2L+ | PBO + BSC | N | ID = 75  Crl = 42 | 1.32  (0.74–2.35) | 0.67  (0.44–1.01) | 43.5 | 31.8 | 30.5 | 15.9 |
| NCT00890825 (17) | NCT00890825 | II | IIIB/IV | MEK inhibitor | Selumetinib + docetaxel | 2L+ | PBO + docetaxel | N | ID = 44  Crl = 43 | 0.80 (0.47–1.37) | 0.58  (0.35–0.93) | 66.7 | 30.0 | 41.3 | 15.0 |
| PRIDE (18) | NCT01196234 | II | IIIB/IV | EGFR TKI | Gefitinib + paclitaxel-carboplatin | 1L | Paclitaxel-carboplatin | N | ID = 44  Crl = 46 | 0.95  (0.58–1.54) | 0.94  (0.61–1.45) | 58.1 | 58.0 | 18.7 | 14.3 |
| PrECOG (19) | NCT00687297 | II | IIIB/IV | VEGFR TKI | Induction docetaxel-carboplatin + vandetanib followed by  vandetanib maintenance | 1L | Induction docetaxel-carboplatin + vandetanib followed by  PBO maintenance | N | ID = 80  Crl = 82 | NA | 1.49  (1.07–2.07) | 60.1 | 51.9 | 38.0 | 25.8 |
| SELECT-1 (20) | NCT01933932 | III | IIIB/IV | MEK inhibitor | Selumetinib + docetaxel | 2L+ | PBO + docetaxel | N | ID = 254  Crl = 256 | 1.05  (0.85–1.30) | 0.93  (0.77–1.12) | 48.1 | 40.0 | 23.0 | 21.5 |
| SELECT-2 (21) | NCT01750281 | II | IIIB/IV | MEK inhibitor | Selumetinib 75 mg BID + docetaxel  60 mg/m^2^ | 2L+ | PBO + docetaxel  75 mg/m^2^ | N | FAS  ID = 85  Crl = 43 | 1.43  (0.91–2.30) | 1.12  (0.75–1.72) | 34.3 | 55.7 | 16.6 | 24.3 |
|  |  |  |  |  |  |  |  |  | *KRAS* wild-type  ID = 15  Crl = 10 | NA | 1.37  (0.28–2.03) | 32.3 | 58.3 | 14.9 | 23.6 |
|  |  |  |  |  |  |  |  |  | No *KRAS*  ID = 62  Crl = 30 | 1.62  (0.91–3.08) | 1.35  (0.82–2.23) | NA | NA | NA | NA |
|  |  |  |  |  | Selumetinib 75 mg BID +  docetaxel 75 mg/m^2^ |  | PBO + docetaxel  75 mg/m^2^ |  | FAS  ID = 84  Crl = 43 | 1.18  (0.74–1.93) | 0.92  (0.61–1.40) | 53.4 | 55.7 | 32.6 | 24.3 |
|  |  |  |  |  |  |  |  |  | *KRAS* wild-type  ID = 19  Crl = 10 | NA | 1.00  (0.47–2.57) | 60.1 | 58.3 | 32.3 | 23.6 |
|  |  |  |  |  |  |  |  |  | No *KRAS*  ID = 54  Crl = 30 | 1.43  (0.78–2.77) | 1.03  (0.60–1.76) | NA | NA | NA | NA |
| ABOUND (22) | NCT02151149 | IV | IIIB/IV | Tubulin inhibitor | *nab*-paclitaxel + carboplatin (21-day arm) | 1L | *nab-*paclitaxel + carboplatin (21-day + break arm) | N | 21-day arm = 71  21-day + break arm = 72 | 0.72  (0.44–1.19) | 0.48  (0.30–0.76) | 82.2 | 52.9 | 71.3 | 40.5 |
| ARCHER 1050 (23) | NCT01774721 | III | IIIB/IV | EGFR TKI | Dacomitinib | 1L | Gefitinib | N | ITT ID = 227 Crl = 225 | NA | 0.59 (0.47–0.74) | 87.9 | 87.7 | 76.5 | 74.7 |
|  |  |  |  |  |  |  |  |  | Asian responders  ID = 131 Crl = 128 | NA | 0.43 (0.32–0.59) | 98.0 | 95.7 | 89.3 | 81.2 |
|  |  |  |  |  |  |  |  |  | Non-Asian responders  ID = 39 Crl = 33 | NA | 0.55  (0.32–0.93) | 89.3 | 90.9 | 70.4 | 72.1 |
|  |  |  |  |  |  |  |  |  | Exon 19 deletion  ID = 134 Crl = 133 | NA | 0.55  (0.41–0.75) | 89.7 | 88.4 | 79.4 | 75.0 |
|  |  |  |  |  |  |  |  |  | L858R mutation  ID = 93 Crl = 92 | NA | 0.63  (0.44–0.88) | 85.6 | 85.6 | 71.5 | 74.2 |
| STUDY 104 (24) | NCT01285609 | III | IV | CTLA-4 inhibitor | Ipilimumab + carboplatin-paclitaxel | 1L | PBO + carboplatin-paclitaxel | N | ID = 479  Crl = 477 | 0.91  (0.77–1.07) | 0.87  (0.75–1.01) | 76.7 | 81.8 | 44.5 | 42.7 |
| PROCLAIM (25) | NCT00686959 | III | IIIA/B | DDR inhibitor | Pemetrexed-cisplatin + TRT  (Arm A) | 1L | Etoposide-cisplatin + TRT  (Arm B) | N | Arm A = 301  Arm B = 297 | 0.98  (0.79–1.20) | 0.86  (0.71–1.04) | 90.3 | 86.8 | 78.4 | 73.4 |
| SQUIRE (26) | NCT00981058 | III | IV | EGFR TKI | Necitumumab + gemcitabine-cisplatin | 1L | Gemcitabine-cisplatin | N | ID = 545  Crl = 548 | 0.84  (0.74–0.96) | 0.85  (0.74–0.98) | 75.5 | 68.9 | 45.0 | 37.0 |
| START (27) | NCT00409188 | III | III | MUC-1 inhibitor | Tecemotide | 2L+ | PBO | N | ID = 829  Crl = 410 | 0.89  (0.77–1.03) | NA | NA | NA | NA | NA |
| INSPIRE (28) | NCT00982111 | III | IV | EGFR TKI | Necitumumab + pemetrexed-cisplatin | 1L | Pemetrexed-cisplatin | N | ID = 315  Crl = 318 | 1.01  (0.84–1.21) | 0.96  (0.80–1.16) | 66.9 | 66.9 | 44.0 | 40.0 |
| NCT00596830 (29) | NCT00596830 | III | IIIB/IV | IGF-1R inhibitor | Figitumumab + paclitaxel-carboplatin | NA | Paclitaxel-carboplatin | N | ID = 342  Crl = 339 | 1.18  (0.99–1.40) | 1.10  (0.93–1.32) | 61.7 | 61.7 | 27.5 | 31.5 |
| NCT00095199 (30) | NCT00095199 | III | III/IV | EGFR TKI | Cetuximab + pemetrexed or docetaxel | 2L+ | Pemetrexed or docetaxel | N | ID = 301  Crl = 304 | 1.01  (0.86–1.20) | 1.03 (0.87–1.21) | 38.2 | 40.7 | 19.5 | 25.6 |
| NCT01836575 (31) | NCT01836575 | III | IIIB/IV | DDR inhibitor | Pemetrexed + carboplatin | 1L | Pemetrexed | N | ID = 102  Crl = 103 | 0.62  (0.46–0.83) | 0.46  (0.35–0.63) | 65.2 | 34.8 | 49.3 | 18.6 |
| PROFILE 1007 (32)* | NCT00932893 | III | IIIB/IV | ALK TKI | Crizotinib | 2L+ | Pemetrexed or docetaxel | Y | ID = 173  Crl = 174 | 1.02  (0.68–1.54) | 0.49  (0.37–0.64) | 75.9 | 47.4 | 58.7 | 31.2 |
|  |  |  |  |  |  |  |  |  |  |  |  | 73.3 | 46.7 | 58.2 | 30.4 |
| NCT01004601 (33) | NCT01004601 | III | IIIB/IV | DDR inhibitor | Cisplatin-pemetrexed | 1L | Cisplatin-gemcitabine | N | ID = 862  Crl = 863 | 0.94  (0.84–1.05) | 1.04  (0.94–1.15) | 59.9 | 63.3 | 34.5 | 37.5 |
|  |  |  |  |  |  |  |  |  | SCC  ID = 244  Crl = 229 | 1.23  (1.00–1.51) | 1.36  (1.12–1.65) | 65.1 | 60.4 | 39.9 | 37.1 |
|  |  |  |  |  |  |  |  |  | Non-SCC  ID = 618  Crl = 634 | 0.81  (0.70–0.94) | 0.90  (0.79–1.02) | 58.4 | 69.8 | 28.2 | 41.1 |
| Pujol et al. (34) | NA | III | IIIB/IV | DDR | Gemcitabine-docetaxel | 1L | Cisplatin-vinorelbine | N | ID = 155  Crl = 156 | 0.90  (0.70–1.16) | 1.04  (0.83–1.32) | 59.6 | 59.6 | 33.8 | 40.2 |
| BR.21 (35) | NCT00036647 | III | IIIB/IV | EGFR TKI | Erlotinib | 2L+ | PBO | N | Young patients  ID = 376  Crl = 192 | 0.73  (0.61–0.89) | 0.64  (0.53–0.76) | 65.3 | 59.7 | 54.4 | 46.1 |
|  |  |  |  |  |  |  |  |  | Elderly patients  ID = 112  Crl = 51 | 0.92  (0.64–1.34) | 0.63  (0.44–0.90) | 64.6 | 57.1 | 52.3 | 44.4 |
| Krzakowski et al. (36) | NA | III | III/IV | Tubulin  inhibitor | Vinflunine | 2L+ | Docetaxel | N | ID = 274  Crl = 277 | 0.97  (0.81–1.18) | 1.00  (0.84–1.20) | 33.3 | 32.2 | 15.3 | 15.3 |
| IFCT-0501 (37) | NCT00298415 | III | IIIB/IV | DDR inhibitor | Carboplatin-paclitaxel | 1L | Gemcitabine or vinorelbine (monotherapy) | N | ID = 225  Crl = 226 | 0.64  (0.52–0.78) | 0.51 (0.42–0.62) | 40.4 | 69.3 | 23.6 | 51.2 |
| East Asia S-1 (38) | JapicCTI-101155 | III | IIIB/IV | Thymidylate synthase inhibitor | Oral 5-fluorouracil-based S-1 | 2L+ | Docetaxel | N | ID = 577  Crl = 570 | 0.95  (0.83–1.07) | 1.03  (0.91–1.17) | 44.1 | 42.7 | 22.8 | 26.3 |
| EURTAC (39) | NCT00446225 | III | IIIB/IV | EGFR TKI | Erlotinib | 1L | Cisplatin/carboplatin-docetaxel or cisplatin/carboplatin-gemcitabine | Y | ITT ID = 86  Crl = 87 | 1.04 (0.65–1.68) | 0.37 (0.25–0.54) | 79.7 | 72.6 | 74.0 | 37.2 |
|  |  |  |  |  |  |  |  |  | *EGFR* mutations detected  ID = 31  Crl = 27 | NA | 0.25 (0.11–0.54) | 75.7 | 70.3 | 71.9 | 37.9 |
|  |  |  |  |  |  |  |  |  | *EGFR* mutations not detected ID = 26  Crl = 25 | NA | 0.29 (0.13–0.63) | 92.0 | 89.4 | 92.0 | 60.9 |
| LETS (40) | NA | III | IIIB/IV | DDR inhibitor | Oral 5-fluorouracil-based S-1 + carboplatin | 1L | Carboplatin-paclitaxel | N | SCC  ID = 55  Crl = 59 | 0.71  (0.48–1.07) | 0.94  (0.64–1.37) | NA | NA | NA | NA |
|  |  |  |  |  |  |  |  |  | Non-SCC  ID = 227  Crl = 223 | 1.06  (0.86–1.31) | 1.06  (0.88–1.28) | NA | NA | NA | NA |
| LUME-Lung 1 (41) | NCT00805194 | III | IIIB/IV | VEGFR TKI | Docetaxel + nintedanib | 2L+ | Docetaxel + PBO | N | ITT ID = 655  Crl = 659 | 0.75 (0.60–0.92) | 0.79 (0.68–0.92) | 43.2 | 32.6 | 22.3 | 17.4 |
|  |  |  |  |  |  |  |  |  | Adeno-carcinoma ID = 322  Crl = 336 | 0.83  (0.70–0.99) | NA | 50.3 | 40.1 | 27.0 | 23.0 |
|  |  |  |  |  |  |  |  |  | SCC ID = 240  Crl = 247 | NA | 0.77  (0.62–0.96) | 38.7 | 24.5 | 20.2 | 11.6 |
| LUX-Lung 6 (42) | NCT01121393 | III | IIIB/IV | EGFR TKI | Afatinib | 1L | Gemcitabin-cisplatin | N | ID = 242  Crl = 122 | 0.95 (0.68–1.33) | 0.28 (0.20–0.39) | 92.6 | 76.0 | 76.3 | 42.5 |
| NCT02737501 (43) | NCT02737501 | III | IIIB/IV | ALK TKI | Brigatinib | NA | Crizotinib | Y | ITT  ID = 137  Crl = 138 | NA | 0.49  (0.33–0.74) | 86.9 | 75.0 | 81.1 | 67.5 |
|  |  |  |  |  |  |  |  |  | CNS mets  ID = 43  Crl = 47 | NA | 0.27  (0.13–0.54) | 95.0 | 66.7 | 87.4 | 46.9 |
| NCT01560104 (44) | NCT01560104 | II | IV | PARP inhibitor | Veliparib + carboplatin-paclitaxel | 1L | PBO + carboplatin-paclitaxel | N | ITT  ID = 105  Crl = 53 | 0.80 (0.54–1.18) | 0.72  (0.45–1.15) | NA | NA | NA | NA |
|  |  |  |  |  |  |  |  |  | SCC  ID = 51  Crl = 25 | 0.73  (0.43–1.24) | 0.54  (0.26–1.12) | NA | NA | NA | NA |
|  |  |  |  |  |  |  |  |  | Non-SCC  ID = 54  Crl = 28 | 0.90 (0.51–1.58) | 0.87 (0.48–1.59) | NA | NA | NA | NA |
| PROFILE 1014 (45) | NCT01154140 | III | IIIB/IV | ALK TKI | Crizotinib | 1L | Pemetrexed + cisplatin or carboplatin | Y | ITT  ID = 172  Crl = 171 | 0.82  (0.54–1.26) | 0.45  (0.35–0.60) | 82.8 | 78.5 | 70.0 | 61.6 |
|  |  |  |  |  |  |  |  |  | Adjusted crossover  ID = 172  Crl = 171 | 0.35  (0.08–0.72) |  |  |  |  |  |
| NCT02075840 (46) | NCT02075840 | III | IIIB/IV | ALK TKI | Alectinib | 1L | Crizotinib | N | ID = 152  Crl = 151 | 0.76  (0.48–1.20) | 0.47  (0.34–0.65) | 81.3 | 80.5 | 73.1 | 76.1 |
| NCT00418886 (47) | NCT00418886 | III | IIIB/IV | VEGFR TKI | Vandetanib + pemetrexed | 2L+ | PBO + pemetrexed | N | ID = 256  Crl = 278 | 0.86 (0.65–1.13)^†^ | 0.86  (0.69–1.06)^†^ | 57.0 | 46.8 | 41.4 | 32.5 |
| NCT00254891 (48) | NCT00254891 | III | IIIB/IV | Toll-Like Receptor 9 Agonist | PF-3512676 + paclitaxel-carboplatin | 1L | Paclitaxel-carboplatin | N | ID = 408  Crl = 420 | 0.95  (0.81–1.12) | 1.02  (0.88–1.19) | 61.3 | 60.2 | 35.3 | 35.1 |
| ZODIAC (49) | NCT00312377 | III | IIIB/IV | VEGFR TKI | Vandetanib + docetaxel | 2L+ | PBO + docetaxel | N | ID = 694  Crl = 697 | 0.91  (0.78–1.07) | 0.79  (0.70–0.90) | 50.9 | 45.1 | 28.7 | 23.0 |
| GEMVIN (50) | NCT00004100 | III | IIIB/IV | Tubulin inhibitor | Cisplatin-containing chemotherapy | 1L | Gemcitabine-vinorelbine | N | ID = 250  Crl = 251 | 1.15 (0.96–1.37)^‡^ | 1.29 (1.10–1.52)^‡^ | 64.7 | 58.5 | 52.9 | 41.9 |
| STUDY BR.18 (51, 52) | NCT00006229 | II | IIIB/IV | Matrix metallo-proteinase  Inhibitor | BMS-275291 + Paclitaxel-carboplatin | 1L | PBO + paclitaxel-carboplatin | N | ID = 38  Crl = 37 | NA | NA | NA | NA | NA | NA |
|  |  | III |  |  |  |  |  |  | ID = 387  Crl = 387 | 1.09  (0.93–1.28) | 0.99  (0.84–1.16) | 62.3 | 64.2 | 43.2 | 43.2 |
| PRONOUNCE (53) | NCT00948675 | III | IIIB/IV | VEGFR TKI | Pemetrexed-carboplatin | 1L | Paclitaxel-carboplatin + bevacizumab | N | ID = 182  Crl = 179 | 1.07  (0.83–1.36) | 1.06  (0.84–1.35) | 60.8 | 71.0 | 37.4 | 42.0 |
| FLEX (54) | NCT00148798 | III | IIIB/IV | EGFR TKI | Cisplatin-vinorelbine + cetuximab | 1L | Cisplatin-vinorelbine | N | ID = 557  Crl = 568 | 0.87 (0.76–1.00) | 0.94 (0.83–1.08) | NA | NA | NA | NA |
| E4599 (55) | NCT03062800 | III | IIIB/IV | VEGFR TKI | Paclitaxel-carboplatin + bevacizumab | 1L | Paclitaxel-carboplatin | N | ID = 434  Crl = 444 | 0.80  (0.69–0.93) | 0.66  (0.57–0.77) | NA | NA | NA | NA |
| BELIEF (56) | NCT01562028 | II | IIIB/IV | EGFR TKI | Erlotinib T790M+  + bevacizumab | 1L | Erlotinib T790M- + bevacizumab | N | ID = 37  Crl = 72 | NA | 0.52  (0.30–0.88) | 94.1 | 88.5 | 85.8 | 78.6 |
| AMG-706 (57) | NCT02629848 | III | IV | NA | Motesanib + paclitaxel-carboplatin | NA | PBO + paclitaxel-carboplatin | N | ID = 197  Crl = 204 | 0.90  (0.62–1.29) | 0.81  (0.64–1.03) | 81.0 | 76.0 | 51.0 | 43.0 |
| LUX-Lung 8 (58, 59) | NCT01523587 | III | IIIB/IV | EGFR TKI | Afatinib | 2L+ | Erlotinib | N | ITT  ID = 398  Crl = 397 | 0.81  (0.69–0.95) | 0.81  (0.69–0.96) | 35.0 | 28.4 | 22.5 | 15.6 |
|  |  |  |  |  |  |  |  |  | TGA results  ID = 132  Crl = 113 | 0.81  (0.62–1.05) | 0.69  (0.51–0.92) | 44.3 | 30.4 | 28.0 | 15.1 |
|  |  |  |  |  |  |  |  |  | Veristat Good = 412  Veristat Poor = 263 | 0.41  (0.35–0.49) | 0.65  (0.54–0.77) | 37.0 | 22.8 | 24.0 | 9.4 |
| GLOB3 (60) | NA | III | IIIB/IV | Tubulin inhibitor | Vinorelbine-cisplatin | 1L | Docetaxel-cisplatin | N | ID = 190  Crl = 191 | NA | NA | NA | NA | NA | NA |
| BMS099 (61) | NCT00112294 | III | IIIB/IV | EGFR TKI | Cetuximab + paclitaxel/ docetaxel-carboplatin | 1L | Paclitaxel/docetaxel-carboplatin | N | ID = 338  Crl = 338 | 0.89  (0.75–1.05) | 0.90  (0.76–1.07) | 49.7 | 48.3 | 29.5 | 25.0 |
| AVAiL (62, 63) | NCT00806923 | III | IIIB/IV | VEGFR TKI | Bevacizumab 7.5 mg/kg + cisplatin-gemcitabine | 1L | PBO + cisplatin-gemcitabine | Y | ID = 345  Crl = 347 | 0.93  (0.78–1.11) | 0.75  (0.64–0.87) | 82.9 | 71.4 | 61.4 | 50.0 |
|  |  |  |  |  | Bevacizumab 15 mg/kg + cisplatin-gemcitabine |  | PBO + cisplatin-gemcitabine |  | ID = 351  Crl = 347 | 1.03  (0.86–1.23) | 0.85  (0.73–1.00) | 74.3 | 71.4 | 56.4 | 52.1 |
|  | NCT01368848 | III | IIIB/IV | VEGFR TKI | Bevacizumab 7.5 mg/kg + cisplatin-gemcitabine | 1L | PBO + cisplatin-gemcitabine | N | Asian subset  ID = 38  Crl = 33 | 0.46 (0.22–0.97) | 0.60 (0.32–1.13) | NA | NA | NA | NA |
|  |  |  |  |  | Bevacizumab 15 mg/kg + cisplatin-gemcitabine |  | PBO + cisplatin-gemcitabine |  | Asian subset  ID = 34  Crl = 33 | 0.79  (0.40–1.57) | 0.65 (0.35–1.22) | NA | NA | NA | NA |
| ZEPHYR (64) | NCT00404924 | III | IIIB/IV | VEGFR TKI | Vandetanib | 2L+ | PBO | N | ID = 617  Crl = 307 | 0.95  (0.81–1.11) | 0.63  (0.54–0.74) | 26.2 | 14.0 | 14.5 | 7.2 |
| EORTC08092 (65)* | NCT01208064 | III | IIIB/IV | VEGFR TKI | Pazopanib | 2L+ | PBO | N | ITT  ID = 50  Crl = 52 | 0.72 (0.40–1.28) | 0.67 (0.43–1.03) | 55.3 | 41.3 | 37.0 | 27.4 |
|  |  |  |  |  |  |  |  |  |  |  |  | 55.6 | 41.2 | 36.6 | 28.0 |
|  |  |  |  |  |  |  |  |  | Squamous  ID = 9  Crl = 11 | 1.98 (0.49–8.05) | 0.38 (0.13–1.12) | 44.3 | 36.6 | 89.2 | 63.7 |
|  |  |  |  |  |  |  |  |  | Non-squamous  ID = 41  Crl = 41 | 0.59 (0.30–1.15) | 0.70 (0.43–1.14) | 57.9 | 42.5 | 35.4 | 28.6 |
| CurrentS (66) | NCT01183858 | III | IIIB/IV | EGFR TKI | Erlotinib 300 mg | 2L+ | Erlotinib 150 mg | N | ID = 160  Crl = 155 | 1.03  (0.80–1.32) | 1.05 (0.83–1.33) | 41.8 | 44.6 | 19.3 | 20.7 |
| IMPower132 (67) | NCT02657434 | III | IV | PD-L1 inhibitor | Atezolizumab + carboplatin/ cisplatin-pemetrexed | 1L | Carboplatin/cisplatin-pemetrexed | N | ID = 292  Crl = 286 | 0.81 (0.64–1.03) | 0.60  (0.49–0.72) | 79.0 | 63.0 | 59.0 | 40.8 |
| Keynote-042 (68) | NCT02220894 | III | IIIB/IV | PD-1 inhibitor | Pembrolizumab | 1L | Pemetrexed/ paclitaxel-carboplatin (SoC chemotherapy) | N | TPS ≥1%  ID = 637  Crl = 637 | 0.81 (0.71–0.93) | 1.07  (0.94–1.21) | 63.0 | 79.0 | 48.0 | 61.0 |
|  |  |  |  |  |  |  |  |  | TPS ≥20%  ID = 413  Crl = 405 | 0.77  (0.64–0.92) | 0.94  (0.80–1.11) | 65.0 | 80.0 | 52.4 | 60.3 |
|  |  |  |  |  |  |  |  |  | TPS ≥50%  ID = 299  Crl = 300 | 0.69  (0.56–0.85) | 0.81 (0.67–0.99) | 65.0 | 77.0 | 55.4 | 59.1 |
| IMpower150 (69) | NCT02366143 | III | IV | PD-L1 inhibitor | Atezolizumab + bevacizumab + carboplatin-paclitaxel | 1L | Bevacizumab + carboplatin-paclitaxel | N | WT population  ID = 356  Crl = 336 | 0.78  (0.64–0.96) | 0.62  (0.52–0.74) | 81.0 | 76.0 | 66.0 | 54.0 |
|  |  |  |  |  |  |  |  |  | Teff-high WT population  ID = 155  Crl = 129 | NA | 0.51  (0.38–0.68) | 84.0 | 71.0 | 71.5 | 56.9 |
| Keynote-189 (70) | NCT02578680 | III | IV | PD-1 inhibitor | Pembrolizumab + cisplatin/ carboplatin-pemetrexed | 1L | PBO + cisplatin/carboplatin-pemetrexed | Y | ITT  ID = 410  Crl = 206 | 0.49  (0.38–0.64) | 0.52  (0.43–0.64) | 78.0 | 63.0 | 66.3 | 40.4 |
|  |  |  |  |  |  |  |  |  | TPS <1%  ID = 127  Crl = 63 | 0.59  (0.38–0.92) | 0.75  (0.53–1.05) | 68.0 | 65.0 | 50.9 | 42.7 |
|  |  |  |  |  |  |  |  |  | TPS 1–49%  ID = 128  Crl = 58 | 0.55  (0.34–0.90) | 0.55  (0.37–0.81) | 81.0 | 74.0 | 71.2 | 41.2 |
|  |  |  |  |  |  |  |  |  | TPS ≥50%  ID = 132  Crl = 70 | 0.42  (0.26–0.68) | 0.36  (0.25–0.52) | 85.0 | 57.0 | 75.0 | 39.5 |
| IMPower131 (71) | NCT02367794 | III | IV | PD-L1 inhibitor | Atezolizumab + carboplatin + nab-paclitaxel | 1L | Carboplatin + nab-paclitaxel | N | ITT  ID = 343  Crl = 340 | 0.88  (0.73–1.05) | 0.71  (0.60–0.85) | NA | NA | NA | NA |
|  |  |  |  |  |  |  |  |  | TC3 or IC3  ID = 47  Crl = 44 | 0.48  (0.29–0.81) | 0.441 (0.25–0.68) | NA | NA | NA | NA |
|  |  |  |  |  |  |  |  |  | TC1/2 or IC1/2  ID = 136  Crl = 125 | 1.08  (0.81–1.45) | 0.70  (0.54–0.91) | NA | NA | NA | NA |
|  |  |  |  |  |  |  |  |  | TC0 and IC0  ID = 160  Crl = 171 | 0.87  (0.67–1.13) | 0.82  (0.65–1.04) | NA | NA | NA | NA |
| OAK (72) | NCT02008227 | III | IIIB/IV | PD-L1 inhibitor | Atezolizumab | 2L+ | Docetaxel | N | ITT  ID = 425  Crl = 425 | 0.73  (0.62–0.87) | 0.95  (0.82–1.10) | 40.0 | 50.0 | 35.1 | 37.6 |
|  |  |  |  |  |  |  |  |  | IC0 (<1%)  ID = 180  Crl = 199 | 0.75  (0.59–0.96) | 1.00  (0.80–1.25) | 36.0 | 50.0 | 26.4 | 27.4 |
|  |  |  |  |  |  |  |  |  | IC1/2/3 (>1%)  ID = 241  Crl = 222 | 0.74  (0.58–0.93) | 0.91  (0.74–1.12) | 44.0 | 51.0 | 31.8 | 29.4 |
|  |  |  |  |  |  |  |  |  | IC2/3 (>5%)  ID = 129  Crl = 136 | 0.67  (0.49–0.90) | 0.76  (0.58–0.99) | 51.0 | 48.0 | 38.4 | 27.4 |
|  |  |  |  |  |  |  |  |  | IC3 (>50%)  ID = 72  Crl = 65 | 0.41  (0.27–0.64) | 0.63  (0.43–0.91) | 53.0 | 45.0 | 41.6 | 22.9 |
| POPLAR (73) | NCT01903993 | II | IIIB/IV | PD-L1 inhibitor | Atezolizumab | 2L+ | Docetaxel | N | ITT  ID = 144  Crl = 143 | 0.73  (0.53–0.99) | 0.94 (0.72–1.23) | 44.0 | 46.0 | 33.2 | 30.4 |
|  |  |  |  |  |  |  |  |  | IC0 (<1%)  ID = 51  Crl = 41 | 1.04  (0.62–1.75) | 1.12  (0.72–1.77) | 41.0 | 51.0 | 26.3 | 32.0 |
|  |  |  |  |  |  |  |  |  | IC1/2/3 (>1%)  ID = 93  Crl = 102 | 0.59 (0.40–0.85) | 0.85  (0.63–1.16) | 47.0 | 42.0 | 36.7 | 29.7 |
|  |  |  |  |  |  |  |  |  | IC2/3 (>5%)  ID = 50  Crl = 55 | 0.54  (0.33–0.89) | 0.72  (0.47–1.10) | 50.0 | 38.0 | 37.8 | 25.1` |
|  |  |  |  |  |  |  |  |  | IC3 (>50%)  ID = 24  Crl = 23 | 0.49 (0.22–1.07) | 0.60 (0.31–1.16) | 67.0 | 41.0 | 57.6 | 27.0 |
| Checkmate-026 (74) | NCT02041533 | III | IV | PD-1 inhibitor | Nivolumab | 1L | Platinum-based chemotherapy | Y | PD-L1 ≥5%  ID = 211  Crl = 212 | 1.02  (0.80–1.30) | 1.15  (0.91–1.45) | 53.0 | 71.0 | 41.8 | 49.5 |
|  |  |  |  |  |  |  |  |  | PD-L1 >50%  ID = 88  Crl = 126 | 0.90  (0.63–1.29) | 1.07  (0.77–1.49) | NA | NA | NA | NA |
| Keynote-010 (75) | NCT01905657 | II/III | IIIB/IV | PD-1 inhibitor | Pembrolizumab | 2L+ | Docetaxel | Y | Pembrolizumab 2 mg/kg  ID = 344  Crl = 343 | 0.71 (0.58–0.88) | 0.88 (0.74–1.05) | 49.0 | 52.0 | 35.2 | 34.1 |
|  |  |  |  |  |  |  |  |  | Pembrolizumab 10 mg/kg  ID = 346  Crl = 343 | 0.61  (0.49–0.75) | 0.79  (0.66–0.94) | 51.0 | 52.0 | 39.5 | 34.1 |
|  |  |  |  |  |  |  |  |  | Pembrolizumab 2 mg/kg;  PD-L1 >50%  ID = 139  Crl = 152 | 0.54 (0.38–0.77) | 0.59 (0.44–0.78) | 60.0 | 54.0 | 47.2 | 32.5 |
|  |  |  |  |  |  |  |  |  | Pembrolizumab 10 mg/kg;  PD-L1 >50%  ID = 151  Crl = 152 | 0.50 (0.36–0.70) | 0.59 (0.45–0.78) | 60.0 | 54.0 | 47.0 | 32.5 |
| Checkmate-057 (76) | NCT01673867 | III | IIIB/IV | PD-1 inhibitor | Nivolumab | 2L+ | Docetaxel | N | ITT  ID = 292  Crl = 290 | 0.73  (0.59–0.89) | 0.92  (0.77–1.11) | 40.0 | 50.0 | 30.6 | 35.0 |
|  |  |  |  |  |  |  |  |  | PD-L1 <1%  ID = 108  Crl = 101 | 0.90  (0.66–1.24) | 1.19  (0.88–1.61) | 26.0 | 48.0 | 22.4 | 32.7 |
|  |  |  |  |  |  |  |  |  | PD-L1 ≥1%  ID = 123  Crl = 123 | 0.59  (0.43–0.82) | 0.70  (0.53–0.94) | 52.0 | 52.0 | 39.3 | 37.5 |
| Checkmate-017 (77) | NCT01642004 | III | IIIB/IV | PD-1 inhibitor | Nivolumab | 2L+ | Docetaxel | N | ID = 135  Crl = 137 | 0.59 (0.44–0.79) | 0.62 (0.47–0.81) | 48.0 | 36.0 | 38.5 | 21.7 |
| JAVELIN Lung 200 (78) | NCT02395172 | III | IIIB/IV | PD-L1 inhibitor | Avelumab | 2L+ | Docetaxel | N | ITT  ID = 396  Crl = 396 | 0.90  (0.75–1.08) | 1.16  (0.97–1.40) | NA | NA | NA | NA |
|  |  |  |  |  |  |  |  |  | PD-L1 ≥1% ID = 264  Crl = 265 | 0.90 (0.72–1.12) | 1.01 (0.80–1.28) | 48.0 | 50.0 | 39.4 | 35.1 |
|  |  |  |  |  |  |  |  |  | PD-L1 >80% ID = 120  Crl = 106 | 0.59 (0.42–0.83) | 0.58 (0.41–0.83) | 58.0 | 37.0 | 49.5 | 26.0 |
| Keynote-024 (79) | NCT02142738 | III | IV | PD-1 inhibitor | Pembrolizumab | 1L | SoC chemotherapy | Y | ID = 154  Crl = 151 | 0.60  (0.41–0.89) | 0.50  (0.37–0.68) | 68.0 | 64.0 | 62.8 | 50.2 |
| Keynote-021 (80) | NCT02039674 | II | IIIB/IV | PD-1 inhibitor | Pembrolizumab + pemetrexed-carboplatin | 1L | Pemetrexed-carboplatin | Y | ID = 60  Crl = 63 | 0.90  (0.42–1.91) | 0.53  (0.31–0.91) | 83.0 | 70.0 | 77.0 | 62.6 |
| CheckMate-227 (81, 82) | NCT02477826 | III | IV | PD-1 inhibitor | Nivolumab + ipilimumab | 1L | Platinum-doublet chemotherapy | N | High TMB (≥10 mutations/ megabase)  ID = 139  Crl = 160 | 0.68  (0.51–0.91) | 0.58 (0.41–0.81)^§^ | 66.0 | 69.0 | 55.6 | 39.9 |
| Keynote-407 (83) | NCT02775435 | III | IV | PD-1 inhibitor | Pembrolizumab + carboplatin-paclitaxel/nab-paclitaxel | 1L | PBO + carboplatin-paclitaxel/nab-paclitaxel | Y | ITT  ID = 278  Crl = 281 | 0.64  (0.49–0.85) | 0.56  (0.45–0.70) | 82.0 | 69.0 | 64.0 | 41.3 |
|  |  |  |  |  |  |  |  |  | TPS <1%  ID = 95  Crl = 99 | 0.61  (0.38–0.98) | 0.68  (0.47–0.98) | 80.0 | 72.0 | 65.6 | 46.7 |
|  |  |  |  |  |  |  |  |  | TPS 1–49%  ID = 103  Crl = 104 | 0.57  (0.36–0.90) | 0.56  (0.39–0.80) | 77.0 | 69.0 | 61.9 | 48.6 |
|  |  |  |  |  |  |  |  |  | TPS >50%  ID = 73  Crl = 73 | 0.64  (0.37–1.10) | 0.37  (0.24–0.58) | 85.0 | 55.0 | 67.0 | 22.9 |
| PACIFIC*  (84–86) | NCT02125461 | III | III | PD-L1 inhibitor | Durvalumab | 2L+ | PBO | N | ID = 476  Crl = 237 | 0.68  (0.53–0.87) | 0.52  (0.42–0.65) | 76.2 | 57.7 | 68.4 | 48.4 |
|  |  |  |  |  |  |  |  |  |  |  |  | 76.0 | 58.0 | 68.00 | 48.5 |

Data from AstraZeneca trials are extracted from the clinical study reports.

*Different analyses/extractions were carried out; ^†^97.58% CI for HR OS and 97.54% CI for HR PFS; ^‡^90% CI; ^§^97.5% CI.

ALK, anaplastic lymphoma kinase; BID, twice daily; BSC, best supportive care; CI, confidence interval; CNS mets, central nervous system metastases; Crl, control; EGFR, epidermal growth factor receptor; FAS, full analysis set; FGFR, fibroblast growth factor receptor; HR, hazard ratio; IC, tumor-infiltrating immune cell; ID, investigational drug; IO, immunotherapy; ITT, intent-to-treat; IV, intravenous; MoA, mechanism of action; NA, not available; NSCLC, non-small-cell lung cancer; OS, overall survival; PBO, placebo; PD-1, programmed cell death 1; PD-L1, programmed cell death ligand-1; PFS, progression-free survival; SCC, squamous cell carcinoma; TC, tumor cell; TGA, tumor genetic analysis; TKI, tyrosine kinase inhibitor; TMB, tumor mutational burden; TPS, tumor proportion score; VEGFR, vascular endothelial growth factor receptor; WT, wild type.

Supplementary Table 2 | Correlation across all trials and by MoA between HR OS and OR PFS4.

| **Label** | **All trials** | **4 major MoAs combined** | **PD-1/ PD-L1** | **EGFR** | **VEGFR** | **DDR** |
| --- | --- | --- | --- | --- | --- | --- |
| Spearman’s Rho* | –0.385 | –0.424 | –0.579 | –0.535 | –0.443 | 0.029 |
| Spearman’s Rho 95% CI, bootstrap | (–0.533;  –0.212) | (–0.581;  –0.241) | (–0.8;  –0.274) | (–0.76;  –0.23) | (–0.993; 0.146) | (–1; 1) |
| Number of drugs | 31 | 20 | 5 | 6 | 5 | 4 |
| Number of trials | 64 | 50 | 16 | 21 | 8 | 5 |
| Number of observations^†^ | 110 | 92 | 38 | 37 | 11 | 6 |
| Slope, meta-regression | –0.156  (–0.233;  –0.079) | –0.172  (–0.255;  –0.089) | –0.192  (–0.28;  –0.104) | –0.23  (–0.344;  –0.116) | –0.125  (–0.356; 0.106) | 0.033  (–0.285; 0.352) |
| Random-effects, meta-regression R^2^ | 10.89% | 14.29% | 72.48% | 35.63% | 0% | 0% |
| Random-effects, meta-regression I^2^ | 69.93% | 68.50% | 16.85% | 61.77% | 12.38% | 88.01% |
| *P*-value | < 0.001 | < 0.001 | < 0.001 | < 0.001 | 0.289 | 0.838 |

*The reported Rho values are negative as an HR <1, and an OR >1, indicate benefit with the investigational product. ^†^Cohort level.

CI, confidence interval; DDR, DNA damage response; EGFR, epidermal growth factor receptor; HR, hazard ratio; MoA, mechanism of action; OR, odds ratio; OS, overall survival; PD-1/PD-L1, programmed cell death-1/programmed cell death ligand-1; PFS4, progression-free survival rate at 4 months; VEGFR, vascular endothelial growth factor receptor.

**Supplementary Table 3 |** Correlation across all trials and by MoA between HR OS and OR PFS6.

| **Label** | **All trials** | **4 major MoAs combined** | **PD-1/ PD-L1** | **EGFR** | **VEGFR** | **DDR** |
| --- | --- | --- | --- | --- | --- | --- |
| Spearman’s Rho* | –0.414 | –0.448 | –0.633 | –0.427 | 0.224 | 0.086 |
| Spearman’s Rho 95% CI, bootstrap | (–0.573;  –0.232) | (–0.619;  –0.248) | (–0.802;  –0.383) | (–0.705;  –0.085) | (–0.638; 0.795) | (–0.92; 1) |
| Number of drugs | 31 | 20 | 5 | 6 | 5 | 4 |
| Number of trials | 64 | 50 | 16 | 21 | 8 | 5 |
| Number of observations^†^ | 110 | 92 | 38 | 37 | 11 | 6 |
| Slope, meta-regression | –0.184  (–0.259;  –0.109) | –0.188  (–0.267;  –0.109) | –0.229  (–0.321;  –0.136) | –0.191  (–0.297;  –0.086) | 0.007  (–0.269; 0.283) | 0  (–0.299; 0.299) |
| Random-effects, meta-regression R^2^ | 23.14% | 24.49% | 86.13% | 36.17% | 0% | 0% |
| Random-effects, meta-regression I^2^ | 66.74% | 65.73% | 9.25% | 61.68% | 12.78% | 88.35% |
| *P*-value | < 0.001 | < 0.001 | < 0.001 | < 0.001 | 0.959 | 0.999 |

*The reported Rho values are negative as an HR <1, and an OR >1, indicate benefit with the investigational product. ^†^Cohort level.

CI, confidence interval; DDR, DNA damage response; EGFR, epidermal growth factor receptor; HR, hazard ratio; MoA, mechanism of action; OR, odds ratio; OS, overall survival; PD-1/PD-L1, programmed cell death-1/programmed cell death ligand-1; PFS6, progression-free survival rate at 6 months; VEGFR, vascular endothelial growth factor receptor.
